# An Alkaline Foregut Protects Herbivores from Latex in Forage, but Increases Their Susceptibility to Bt Endotoxin

**DOI:** 10.3390/life13112195

**Published:** 2023-11-11

**Authors:** Vidya Rajan

**Affiliations:** Department of Medical and Molecular Sciences, University of Delaware, Newark, DE 19716, USA; vidyarajan@hotmail.com

**Keywords:** latex, ruminants, alkaline gut pH, foregut, hindgut, monogastric, polygastric, herbivory, deterrent, antifeedant, evolution, digestive system, adaptation, *B. thuringiensis*, Bt toxin, bioinsecticide

## Abstract

About 10% of angiosperms, an estimated 20,000 species, produce latex from ubiquitous isoprene precursors. Latex, an aqueous suspension of rubber particles and other compounds, functions as an antifeedant and herbivory deterrent. It is soluble in neutral to alkaline pH, and coagulates in acidic environments. Here, I propose that foregut-fermenting herbivores such as ruminants, kangaroos, sloths, insect larvae, and tadpoles have adapted to latex in forage with the evolution of alkaline anterior digestive chamber(s). However, they consequently become susceptible to the action of *Bacillus thuringiensis* (Bt) δ-endotoxin and related bioinsecticides which are activated in alkaline environments. By contrast, hindgut-fermenting herbivores, such as horses and rabbits, have acidic anterior digestive chambers, in which latex coagulates and may cause gut blockage, but in which Bt is not activated. The latex-adapted foregut herbivore vs. latex-maladapted hindgut herbivore hypothesis developed in this paper has implications for hindgut-fermenting livestock and zoo animals which may be provided with latex-containing forage that is detrimental to their gut health. Further, ruminants and herbivorous tadpoles with alkaline anterior chambers are at risk of damage by the supposedly “environmentally friendly” Bt bioinsecticide, which is widely disseminated or engineered into crops which may enter animal feed streams.

## 1. Introduction

The ability to extract energy from food is essential for animals [1]. Many obligate herbivores obtain their energy from angiosperms whose recalcitrant cell wall constituents, cellulose and lignin, retard digestion, and which may additionally contain antifeedants such as latex, resins, gums, silicates, phenolics, and alkaloids. Herbivores include nematodes; some species of molluscs and crustaceans [2]; the larvae and adults of many insects; the tadpoles of some anurans; certain fish, birds, reptiles; and odd- and even-toed ungulates and primates among mammals. This variety obscures similar behavioral adaptations such as avoidance of noxious plants and parts, and physiological adaptations such as the presence of salivary proteins for detoxification and digestion [3], and fermentation chambers with symbionts to assist the digestive process [4].

Plants produce a slew of chemicals as deterrents to herbivory (Supplementary Table S1). Although latex functions to plug wounds in the plant body, latex is primarily considered to be an antifeedant [5]. Latex is an emulsion with suspended particles including rubber, and may additionally contain other deterrent metabolites such as cardiac glycosides in milkweed and alkaloids in the opium poppy [6]. Rubber is a megapolymer of terpene units, which is sequestered in particles with a protein-rich membrane. When exposed to acid, the membrane is removed and the rubber inside is released like strands of spaghetti to form insoluble tangles [7]. Latex remains liquid in neutral and alkaline environments because the membrane is not disrupted. The exact timeline of the evolution of latex is uncertain, but the major precursor for latex is the common isoprene molecule whose use spans a spectrum from the synthesis of fatty acids for essential structural membrane components to secondary metabolites that provide evolutionary advantage, all the way to terpene compounds with antifungal, anthelminthic, antibacterial, cytotoxic, and insect-repellent properties [5,8,9].

Latex-containing plants span 43 families and 20,000 species, and make up a large proportion (>10%) of angiosperms, one fern (*Regnellidium diphyllum*) and one gymnosperm (*Gnetum gnemon*) [8]. They overlap with areas with enormous herbivore diversity in the tropics and have a variety of uses for humans as well. Some are cultivated for food [5], rubber [10], pharmacologically active molecule production [8], and herbivore-deterrent fencing [11]. Despite clear evidence of latex’s deterrence of herbivory, and the abundance and diversity of plants that produce latex, the question of how the two major categories of herbivores, foregut fermenters and hindgut fermenters, tackle the small or large amounts of latex that must inevitably enter their diet has not been explored, even by the venerable 454-page volume, The Ecology of Browsing and Grazing II, in which the word “latex” does not even appear [12]. And with paper titles like Grazers and Browsers: How Digestive Morphology Affects Diet Selection [13], the prevalent assumption is that gut morphology, and, presumably, associated physiology shapes forage selection, rather than the other way around. Many plants’ latexes, such as from papaya, *Ficus* species, dandelions, mulberry, and the rubber tree lack toxicity, and the assumption has been that its sticky nature, which can gum up mouthparts and retard mobility, is alone responsible for the deterrent effect on herbivory [14]. Konno et al. (2004) even state, “*However, the absence of apparent toxicity from such plants appears to be inconsistent with and even undermining the widely accepted defense hypothesis.*” [15]. I propose, instead, that the deterrent effect of latex lies in the variable behavior of rubber particles in the latex in the alkaline or acidic conditions encountered in the anterior digestive chambers of foregut and hindgut fermenters, respectively.

### 1.1. Latex as a Herbivory Deterrent

Studies focused on selection pressures on the evolution of herbivory have noted that herbivore pressure on plants increases towards the Equator, and plants respond with a higher density of defense towards generalist herbivores [16]. The notion that angiosperms radiated from the Cretaceous is well accepted [17], and it is also accepted that “*Because some of the oldest and most diverse angiosperm floras are found in Africa near the Equator, followed by low-latitude, angiosperm-dominated floras in North America, angiosperms are thought to have radiated from the Equator and spread to either pole*.” [18].

Complementary studies have identified latex and its component molecules as deterrents for herbivory. A study of the common dandelion (*Taxaracum officinale*) showed that latex fouls chewing mouthparts and that a constituent secondary metabolite, the sesquiterpene lactone taraxinic acid β-D-glucopyranosyl lactone (TA-G), has a marked effect on the fitness of larvae of the Coleopteran May bug beetle, (*Melolontha melolontha*) [19]. Even the Monarch butterfly (*Danaus plexippus*) caterpillars, which have been specialized to feed on milkweed (*Ascelpias* spp.), avoid consuming latex, severing latex-carrying tubes and feeding on the latex-depleted portions of leaves [14]. In a separate experiment, washing off latex from fig leaves made the leaves more palatable to herbivores [15]. Latex may vary in composition, presumably due to regional selection pressures the plants experience, but the hypothesis that latex is a herbivory deterrent has gained wide acceptance [5].

### 1.2. Divergent Digestive Morphology of Foregut- and Hindgut-Fermenters

**Figure 1:**
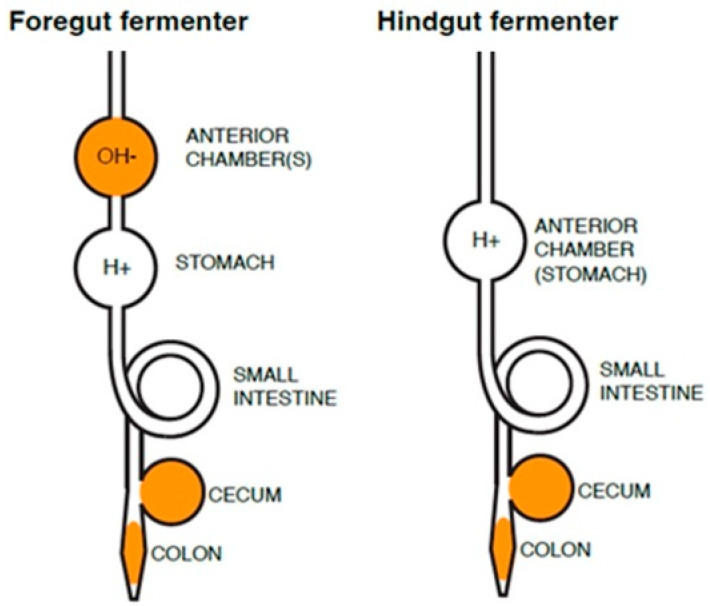
Schematic of location of symbiont-mediated fermentation chambers in mammalian herbivores’ gastrointestinal tracts. Foregut fermenters have neutral- to alkaline-pH chambers (OH^−^) anterior to the acidic stomach (H^+^) and may also have cecum- and/or colon-associated symbionts, whereas hindgut (cecum and colon) fermenters have anterior acidic stomach chambers and cecum- and/or colon-associated symbionts. Regions where symbionts may reside are shown in solid color.

Foregut-fermenting herbivores consume latex-containing shrubs, forbs, and leaves as well as latex-free grasses, and have multiple (polygastric) chambers with neutral-to-alkaline pH in which fermentative symbionts occur, and which are located anterior to the acidic stomach (abomasum). Foregut-fermenting animals include ruminants with a four-chambered digestive system and which regurgitate and chew cud (deer, goats, cows, sheep) and nonruminants which do not regurgitate cud (hippos, kangaroos and sloths) [19]. All ruminating mammals fall in the order Artiodactyla (even-toed ungulates; also classified as order Cetartiodactyla). By contrast, hindgut-fermenting herbivores, such as horses, rabbits, elephants and rhinos, are monogastric animals with an anterior acidic stomach, and a posterior cecum or colon—the location(s) where symbiont-assisted fermentation occur(s). Hindgut-fermenting mammals largely belong to the order of Perissodactyla (odd-toed ungulates) or Proboiscidea (elephants) [20]. Figure 1 shows a schematic of the location of anterior and posterior chambers in mammals that have foregut or hindgut (cecal, colon) fermentation.

Smaller herbivores, such as insect larvae and tadpoles, lack acidic chambers in the digestive system. Insects have cuticle-lined foreguts and hindguts, with the midgut providing digestive and absorptive functions and characterized by neutral to highly alkaline milieus [21]. Captive tadpoles of the model organism *Xenopus laevis* are fed pellet food which includes algae and shrimp flakes [22], and their gut is reported to be structurally similar to the mammalian gut [23]. However, stomachs of tadpoles do not secrete acid [24]. Thus, while there may be an anatomical resemblance to mammalian guts, it does not translate to a functional resemblance.

### 1.3. Alkaline Guts Show Susceptibility to Bacillus thuringiensis and Related δ-Endotoxins

An alkaline gut, however, renders animals susceptible to the action of toxins produced by *Bacillus thuringiensis* and *Lysinibacillus sphaericus* (previously *Bacillus sphaericus*) [25]. These are Gram-positive bacilli which produce sporulation-associated crystal (Cry) δ-endotoxin, cytolytic (Cyt), Mtx (mosquitocidal), and Bin (binary) toxins. During vegetative growth they produce vegetative insecticidal protein (Vip) and secreted insecticidal protein (Sip) [26]. *B.thuringiensis* Cry δ-endotoxins (Bt) are considered as target-specific biocontrol agents for the larvae of many invertebrate pest species, largely among Lepidoptera and Diptera, but also Coleoptera and Nematoda. Bt toxins are solubilized in an alkaline environment as a prerequisite to their proteolytic activation. Activated toxin inserts into gut membranes to form pores, and causes loss of structural integrity in environments such as insect guts [27] but potentially also in foregut-fermenting ruminants and tadpoles. Bt toxins have also been genetically engineered into a variety of important food and cash crops such as corn, soybeans, eggplants, and cotton, which may enter animal-feed streams. Additionally, Bt toxins’ reputation as an environmentally friendly biocontrol agent with few reported off-target effects has led to its widespread dissemination in the environment, including into water bodies. The association of susceptibility of foregut-fermenting herbivores to this type of toxin has not been made before.

### 1.4. Latex in Forage May Have Shaped the Morphology of Animal Digestive Systems

There are three correlations proposed in this paper to support the selective pressure of latex as the factor that shaped the evolution of digestive systems. Although direct evidence will have to be collected to assess these correlations for latex tolerance, there is circumstantial evidence for the following that support latex-based selection:

1. Areas with abundant latex-containing plants correlate with the origins of foregut-fermenting animals. This correlation indicates that latex may have exerted a selective pressure for gut alkalinity.
2. Foregut-fermenting (often polygastric) animals have higher first-chamber pH than hindgut-fermenting (monogastric) animals. This allows the former to tolerate small amounts of latex in the diet, whereas the latter have to avoid latex-containing forage. Awareness of this correlation is important anywhere captive animals are provided with forage, such as in farming operations or zoos.
3. The presence of an alkaline gut pH makes foregut-fermenting vertebrates, metamorphosing tadpoles, and certain orders of insects susceptible to gut damage from Bt δ-endotoxin and related insecticidal toxins. Due to their perceived target-specific nature, these toxins have been widely disseminated and may pose a health threat to foregut-fermenting animals in the wild, as well as to domesticated livestock.

The purpose of this study is to demonstrate that herbivores, ranging from insect larva and tadpoles to ruminants, could evade the deterrent action of latex by the presence of alkaline pH in the anterior gut chambers. Alkaline environments maintain latex in liquid form, thus avoiding gut blockage. However, this physiological protection against latex may render such herbivores susceptible to the action of the widely applied Bt toxins. There is little research into the examination of the evolution of the two different types of herbivore gut architecture, and it is hoped that this paper will stimulate more research into latex as a selective pressure for evolution of herbivore anatomy and physiology.

## 2. Materials and Methods

### 2.1. Literature Search

A literature search was performed using the Google Scholar search engine (scholar.google.com) and the checklist from PRISMA-S (Preferred Reporting Items for Systematic reviews and Meta-Analyses literature search extension) [28]. Searches for articles for the categories were conducted between August 2023 and October 2023 using the terms listed below. English was the search language and other language articles were excluded, but no other limits were applied. In Google Scholar, papers were sorted “by relevance”, and “any type” of articles, except Case Law, were allowed. Each search term pulled up many tens of thousands, and sometimes even hundreds of thousands of titles, so the search processes was standardized to identify the most relevant papers as described below.

For the first page of results (10 titles) which were algorithmically assessed as of highest relevance to the search terms, the abstracts were perused. If the abstracts were judged to be interesting, the full article was obtained from Google Scholar, the University of Delaware library, ResearchGate.net, or the Internet Archive (archive.org (accessed on 5 November 2023)). For each article that was judged relevant, “Related articles” were scrutinized by title and abstract as described above. When available, PDFs of articles were searched using “Find text in document” function for specific terms of interest, such as alkaline, latex, symbionts, to quickly establish relevance. The reference list provided in articles of interest was accessed when in-text citations were of interest. In these cases, the article was obtained and the abstract was assessed for relevance.

### 2.2. Search Categories

1. Stomach pHs of foregut- and hindgut-fermenting herbivores.

Search terms: Stomach pH levels; stomach acidity levels; foregut pH; hindgut pH; herbivore stomachs; polygastric monogastric stomachs; and foregut hindgut herbivores.

2. Plant deterrents against herbivory, including latex.

Search terms: herbivory deterrent; latex deterrent; latex plants herbivory; plant antifeedants; and secondary metabolite latex deterrents.

3. Geography and evolution of foregut and hindgut fermenters.

Search terms: evolution foregut hindgut; evolution of digestive systems herbivores; where did herbivores evolve; evolution Perissodactyla; evolution Artiodactyla; and evolution Cetartiodactyla.

4. The action of *Bacillus thuringiensis* and related toxins in animals with alkaline gut pH.

Search terms: Bacillus toxin effect animals; Bt alkaline gut; Bt effect ruminants; and Bt mode of action.

5. Occasionally, search terms were used to answer specific questions that arose while writing the paper. They were: stomach pH (wild animals by name); hindgut foregut digestion Cretaceous; evolution Artiodactyla Cretaceous; latex levels mature young leaves; Bt effect tadpoles; horses unripe apples; and cattle silvopasture.

### 2.3. Data Analysis

There is a paucity of information on the phylogeny of foregut- and hindgut-fermenting herbivores. I sorted the information available with the purpose of correlating evolutionary origins of herbivores to geographical locations of origin. This search did not yield unambiguous results, and so was not pursued further.

After obtaining data on the stomach pHs of herbivores, I tabulated the existing classifications of the animals as foregut- or hindgut-fermenting herbivores. Only one animal, the quokka, was mis-classified in one of the references. Other problems that arose were mean pH being cited in reviews as representative of whole chamber pH (pointed out by a reviewer). Therefore, original work was checked and the values for anterior chamber (cardiac stomach rather than pyloric stomach) pH (rather than mean chamber pH) were obtained where available. pH values were rounded to one decimal place. Averages were taken when ranges were reported.

Then, I sorted the data by pH level and produced a graphic form of the range of pH and the two types of digestive systems for better visualization of the pH difference between the two groups. The mean of the group’s values shown in Table 1 (foregut fermenters) and Table 2 (hindgut-fermenters) was calculated using https://www.socscistatistics.com/tests/studentttest/default2.aspx (accessed on 5 November 2023). The calculator on the website was used to calculate the independent two-tailed Student’s *t*-test values for a significance of *p* < 0.05. The standard deviation (SD) was calculated using https://www.calculator.net/standard-deviation-calculator.html (accessed on 5 November 2023). The graph was drawn using Microsoft Excel Microsoft 365 MSO (Version 2310 Build 16.0.16924.20054) 64-bit under a personal license.

The popular biocontrol agent, *Bacillus thuringiensis* δ-endotoxin and related insecticidal toxins, are solubilized by alkaline gut pH as a prerequisite to their activation by proteases. Therefore, I further explored the literature for the impact of crystal toxins on ruminants and metamorphosing herbivores such as tadpoles, which have alkaline gut pH as larvae but become carnivores with acidic stomachs as adults.

## 3. Results and Discussion

1. Areas with abundant latex-containing plants coincide with the regions of origin of foregut-fermenting animals.

Foregut and hindgut herbivores, insects, and tadpoles have dispersed from their areas of origin due to natural and human-mediated actions, so it is not a straightforward matter to assess the pressures extant during the evolution of the various species, or to delineate the complex interactions influenced by climate, competition, and biogeography between plants and herbivores. However, since latex-bearing plants are most abundant in the tropics, which correlates with the greatest herbivore pressures [17], latex providing the selection pressure for the evolution of alkaline guts is a reasonable inference.

It is thought that the mammalian order Perissodactyla (odd-toed ungulates; monogastric animals; and hindgut fermenters) evolved in North America, Europe, and Asia, all of which were part of the Laurasia supercontinent during the pre-Cretaceous when flowering plants evolved, diversified, and spread [29]. However, perissodactyl animals would not have been exposed to latex in their diet. Their presence in Africa and South America is due to migration from Europe and North America [30]. Most hindgut fermenting are monogastric grazers which eat grasses and have dentition to break down silica-containing highly abrasive plant material. Their forage diversity is limited and poor in nutrition. Therefore, they have to eat unremittingly and become extremely large to accommodate the passage of the large quantity of low-quality forage they eat (elephants, rhinos, horses) or practice coprophagy (rabbits).

The evolution of artiodactyls is thought to have been more recent, in what is today’s Pakistan in the Eocene epoch (56–33.9 Ma) of the Cenozoic era, and they spread out during the Oligocene (33.9–23 Ma). By the Pliocene (5.3–2.6 Ma), artiodactyls were established in South America [31]. These animals had/would evolve foregut-fermenting digestive systems. These polygastric animals have a herbivory-adaptable digestive system due to the presence of foregut fermentation, as well as cecum fermentation to break down recalcitrant molecules. Old world monkeys (langur, colobus) and new world monkeys (spider) are foregut fermenters and they live in tree canopies and eat leaves which may contain latex. Red howler monkeys also live in tree canopies but have a diverse diet with little latex, so their hindgut digestive system is adequate for their needs [32].

In addition to the evolution of alkaline anterior digestive chambers as an adaptation to latex, it is a reasonable inference that foregut fermenters evolved in and occupy a wider diversity of habitats from tree canopies to woodland fringe to pasture, whereas hindgut fermenters occupy grassland or selectively consume latex-free plants in regionally restricted habitats. Significantly, carnivores, omnivores, and herbivores with hindgut fermentation and associated acidic stomachs are largely capable of pivoting to new diets, provided the new diet is latex-free. This was seen in a population of Italian wall lizards (*Podarcis sicula*) of which a few breeding pairs were transported from the island of Pod Kopište where they had an insectivorous diet to the island of Pod Mrčaru where, in a mere 36 years, they adapted to a largely herbivorous diet [33]. This habitat pivot, according to the hypothesis presented in this paper, would not have been possible had the forage consisted of mostly latex-producing plants.

Although this is a scattershot justification rather than prediction, the correlations still apply. Interestingly, the giant panda is an exception to this hueristic. Its forage is extremely protein-rich bamboo shoots and it has carnivore-like digestion with neither foregut nor hindgut fermentation [34].

2. Foregut-fermenting browsers have higher first-chamber pH, predictive of higher latex tolerance, than hindgut fermenters.

Fresh latex from plants is liquid and has a pH between 6–7.5 [35]. The neutral-to-alkaline pH of foregut herbivores is capable of maintaining latex in liquid form. The large gut capacity of ruminants further provides a dilution factor, so rubber particles are more dispersed and less likely to occlude the system. Ruminants regurgitate and chew the cud to break up clumps, and have symbionts to digest recalcitrant molecules such as cellulose, and possibly rubber as well. Although the presence of rubber-digesting symbionts from foregut-fermenting herbivores has not been reported in the literature, rubber-degrading actinomycetes in the genus *Gordonia* have been isolated from *Hevea* tree bark and the environment [36] and are potentially present in environments where they may be ingested by animals feeding on latex-bearing plants.

To summarize, foregut-fermenting animals which eat leaves and forbs as well as grasses appear to be able to handle small amounts of latex in their mixed forage without adverse effects. Smaller animals like caterpillars avoid ingesting latex by exhibiting latex-avoidance behaviors like trenching and vein cutting [14], but, given their extremely small size, even tiny amounts can cause gut blockage if it solidifies. Therefore, the presence of an alkaline gut, which keeps latex liquid, is even more critical. Table 1 shows the pH levels of an assortment of foregut fermenters. Where the pHs of cardiac and pyloric regions were measured, the cardiac portion of the stomach’s pH was selected for the table.

**Table 1:** First chamber pHs of foregut-fermenting herbivores.

Common Name	Binomial Name	Anterior Chamber pH	Reference
Ox	*Bos* sp.	6	[37]
Hippo	*Hippopotamus amphibius*	5.7	[38]
Sheep	*Ovis aries*	6.4	[37]
Brocket deer	*Mazama* sp.	6.5	[39]
Langur monkey	*Presbytis cristatus*	5.9	[40]
Collared peccary	*Tayassu pecari*	6.3	[39]
Cows	*Bos taurus*	6.1	[41]
Colobus monkey	*Colobus polykomos*	6.8	[42]
Camel	*Camelus* sp.	6.4	[43]
Hoatzin bird *	*Opistocomus hoazin*	6.4	[44]
Macropodid kangaroo	Macropodidae	6.9	[45]
Guanaco	*Lama guanicoe f. glama*	6	[46]
Sloth	*Choloepus* sp.	7.4	[45]
Quokka **	*Setonix brachyurus*	6.8	[47]
Flathead grey mullet	*Mugil cephalus, M.cerama*	7.8	[48]

* The hoatzin is the only known bird folivore with foregut fermentation; ** The quokka is a ruminant according to [47].

Animals which are likely to encounter substantial amounts of latex on a daily basis such as sloths which feed on the latex-containing *Ficus* tree [49], and colobus monkeys which feed largely on leaves but also fruit when available [50], have a relatively high pH in the first chamber (Table 1: sloths = pH 7.4; colobus monkeys = pH 6.8), whereas foregut-fermenting hippos and sheep are largely grass grazers and have a slightly lower anterior chamber pH [Table 1; hippos-pH = 5.7; sheep = pH 6.4]. This observation indicates a direct relationship between the amount of latex in the diet and levels of alkalinity in the anterior chamber. Another important caveat to these values is the variability of pH during a single day and pre- and post-prandial sampling. These variations are not part of the record; pH values also varied by sampling technique [41]. Thus, the values in the table should be treated as indicators of chamber pH, but not as absolute values.

Table 2 shows the anterior digestive chamber pH of hindgut-fermenting animals which primarily eat latex-free forage, including grains, fruits, nuts, and sometimes carrion. The presence of an acidic stomach would hinder hindgut fermenters from tolerating even moderate amounts of latex in the diet. An acidic first chamber may serve as a barrier against pathogens ingested with the oral route due to the largely grazing habitat increasing the potential for consuming feces deposited by other animals, thereby ingesting pathogens and parasites. Additionally, grains and nuts contain high levels of proteins which are broken down by proteases in the presence of stomach acid.

**Table 2:** Anterior chamber pH of hindgut-fermenting herbivores.

Common Name	Binomial Name	Anterior Chamber (Stomach) pH	Reference
Beaver	*Castor canadensis*	1.6	[51]
Rabbit	*Oryctolagus cuniculus*	1.9	[37]
Elephant	*Loxodonta africana*	3.1	[38]
Rhino	*Diceros bicornis*	4.5	[38]
Southern hairy nosed wombat	*Lasiorhinus latifrons*	3.3	[52]
Guinea pig	*Cavia porcellus*	4.5	[37]
Horse	*Equus ferus caballus*	5.4	[37]
Howler monkey	*Alouatta palliata*	4.5	[53]
New world porcupine	*Erethizon dorsatum*	4.5	[54]
Gerbil	Gerbillinae	5.5	[37]
Golden hamster	*Mesocricetus auratus*	5.1	[55]

Latex-feeding studies in hindgut fermenters have not been reported. Remarkably, however, there is documentation of a woman (humans are hindgut fermenters) who ingested liquid latex. The latex solidified into a solid mass, which the surgeons attributed to its exposure to stomach acid, took on the shape of the stomach and completely blocked it. It reportedly bounced like a rubber ball upon surgical removal [56].

**Figure 2:**
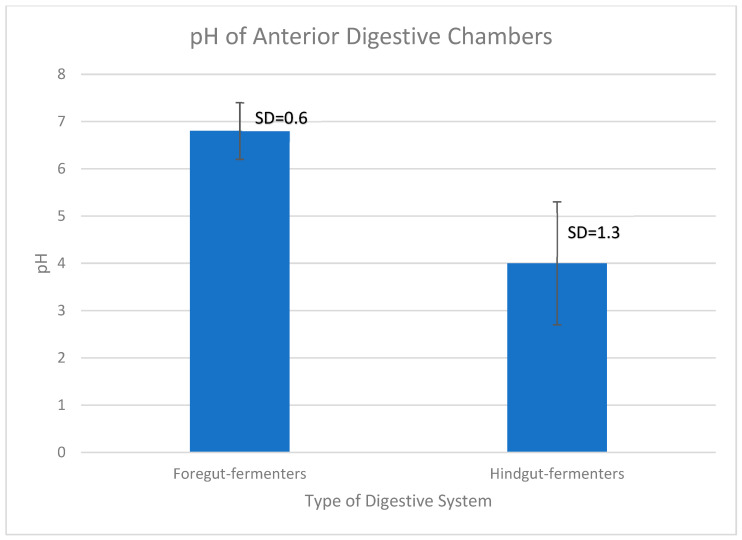
Average anterior-chamber pH of foregut and hindgut fermenters. There is a ~2.8 point difference between the avearge pH of the first digestive chamber of foregut (pH = 6.8) and hindgut fermenters (pH = 4). Data were taken from Table 1 and Table 2. The bars indicate the standard deviation (SD). An independent two-tailed Student’s *t*-test was performed using the calculator on the site https://www.socscistatistics.com/tests/studentttest/default2.aspx (accessed on 5 November 2023). The *t*-value is 6.51328. The *p*-value is <0.00001. The result is significant at *p* < 0.05. Standard deviation was calculated using https://www.calculator.net/standard-deviation-calculator.html (accessed on 5 November 2023), and graphed using Excel.

In order to assess if the pH values between foregut and hindgut fermenters’ first chambers were significantly different to support the conjectures above, an independent two-tailed Student’s *t*-test was conducted. The graph in Figure 2 shows that there is a significant difference in the average chamber pH between the groups of animals whose anterior chamber pH is listed in Table 1 and Table 2.

3. The presence of an alkaline gut pH makes foregut-fermenting mammals, metamorphosing tadpoles, and certain orders of insects susceptible to gut damage by Bt and related insecticidal toxins.

While direct measurement may be difficult in very small animals, there is an indirect way to test the pH of the first chamber of the gut by measuring the organism’s susceptibility to *Bacillus thuringiensis* crystal δ-endotoxin (Bt). Bt toxins are proteins which are solubilized and proteolytically activated in an alkaline pH [57]. When activated, they punch holes in the gut lining, causing leakage and death [27]. If the proteins encounter acid in the first chamber, they remain in an inactive form and are degraded by proteases, and thus made innocuous.

Larvae of Lepidoptera (moths and butterflies), Coleoptera (beetles), Diptera (flies and mosquitoes), Hemiptera (true bugs), and Hymenoptera (bees, wasps and ants) typically have neutral to alkaline midguts, and many include plant material in their diets. The larvae are susceptible to Bt toxins, which are used as biocontrol agents. Coleoptera larvae, which have a slightly more acidic to neutral midgut (pH 5–7), are less susceptible than Lepidoptera and Diptera larvae which have a more alkaline midgut (pH  >  9) [58]. Interestingly, a data point that Huber et al. (2016) could not explain in their study of the Coleopteran larvae of May bugs feeding on dandelion roots was that the larvae gained mass when they fed on latex, even as the concentration of the secondary metabolite TA-G in the latex negatively correlated with *M. melolontha* growth [18]. This observation can be explained if the latex was solidifying inside the larva due to Coleopterans’ more acidic gut pH, and, therefore, failing to be digested and eliminated, resulting in the observed increase in mass.

The presence of alkaline guts in foregut fermenters has significant implications for the use of insect larvicidal toxins for biological control. In sporadic reports, Bt was claimed to be toxic to cows, goats, buffaloes, and sheep [59,60,61], which would support the hypothesis of the alkaline pH of their guts activating the toxin. The presence of alkaline gut chambers has not been addressed before in the scientific literature as a causative agent for Bt-engineered plants and their products being detrimental to foregut herbivores. Instead, other reports have focused on allergenicity and leakage of toxins into the bloodstream or milk of cows as pathways by which Bt might cause damage, and little has been done to examine the long-term effect of Bt in ruminant feed [62]. Silaging appears to remove the toxic effect, consistent with the process of breaking down Bt toxin [63,64].

In an experiment conducted by Lajmanovich et al. (2015), Bt proved toxic to the tadpoles of the South American common frog, *Leptodactylus latrans* [65]. Herbivorous tadpoles have a stomach with a neutral pH, but, as these tadpoles metamorphose to adulthood and obligate carnivory, their stomach pH changes to acidic [66]. Although not all gut pHs have been measured, an indirect measurement can be applied by assessing susceptibility to Bt toxins which, as mentioned before, requires alkaline pH for solubilization followed by proteolytic activation. Lajmanovich et al., 2015 [65] did not extend their findings to foregut-fermenting herbivores. A master’s dissertation by Zaayman (2012) also documented the possibility of Bt in maize leaves as a stressor during *Xenopus laevis* tadpole development, but handling and other complications diluted the clarity of the effect [67].

Thus, animals with an alkaline first chamber will both activate Bt toxin and not cause latex to coagulate, whereas animals with an acidic gut will break down crystal toxins, but also cause latex to coagulate. In the presence of both latex and Bt, gut blockage or gut leakage are the options on offer.

## 4. Implications

The primary hypothesis presented in this paper is that the neutral-to-alkaline pH of the anterior digestive chambers of foregut-fermenting herbivores has a straightforward evolutionary purpose: to enable tolerance of the widespread plant metabolite, latex. Obviously, this does not preclude the adaptation of the chamber to symbionts, since the moderate pH is amenable to supporting a variety of organisms. This hypothesis is further supported by the presence of neutral-to-alkaline anterior digestive chambers in folivores, which eat leaves with high levels of latex, and frugivores, where latex is a gatekeeper for fruit ripeness (see Table 1). Mature leaves and ripe fruit typically have lower latex levels, and are more often browsed than tender shoots [68]. Some plant latexes, like papaya latex, do not contain toxic chemicals but show high levels of proteases which may provide an additional deterrent to herbivory [15]. Thus, a secondary hypothesis emerges that toxic compounds, where they exist in latex, are directed towards foregut-fermenting herbivores which can tolerate latex ingestion; but latex-producing plants would prefer to not be eaten at all, and so produce still other deterrents to herbivory (see Supplemental Table S1).

The choice of food for animals that are confined, as in zoos or domesticated or not able range on their own, is critical for their health. In zoos, care is taken to monitor nutrition, and usually zoos follow the precedents of other zoos which have successfully nurtured a picky herbivore. But it is as well to know why, so that the diet can be modified to resemble the wild diet [69], but within the bounds of the capacity of the animal’s digestive system. Domesticated herbivores are also at risk if food is offered without foresight. Apples and persimmons, among many other fruits, contain latex as a ripeness gatekeeper, and may be commonly planted in pastures. It is known that horses should not eat a lot of unripe apples. The National Equine site has the following comment: *Yes, horses can eat unripe apples. However, horses **prefer** ripe apples because they are sweeter and easier to eat*. *Unripe apples can be **quite tart** and might not be enjoyable for horses”*. [70]. The bolding in the preceding sentences represents my emphasis; the “preference” may be due to other things besides “tartness”, which may not be appreciated without an understanding of the role of latex. Since horses are hindgut fermenters with acidic first chambers, latex in feed will coagulate rather than break down, potentially causing obstruction and pain. Regarding foregut-fermenting cattle, a recent and highly successful move to silvopasture where animals are released into lightly forested woodland can be attributed to their being returned to the environment in which they evolved to eat grasses, forbs, and leaves [71]. Foregut-fermenting bison and deer are notorious for eating young saplings and preserving and producing grasslands, but they can cope with the latex produced by those very saplings. Horses do not forage on trees. Such observations can now be put into a physiological context.

Another interesting consequence of alkaline guts as protection against latex, relates to the widespread use of Bt and similar protein biopesticides which are thought of as highly target-specific. Although Bt’s safety as an ecologially responsible insecticide has devoted champions [72], it is not clear that the distinction between digestive modes of different animals was taken into consideration. Rather, safety tests were carried out on non-target insects and laboratory animals such as hindgut-fermenting rats and mice [73], making Bt’s off-target impact and consequent ecological safety questionable. Specifically, the effect of Bt on alkaline-gut vertebrates, such as ruminants and tadpoles was not considered in assessing the safety profile. By using the metric that these proteins are activated in an alkaline gut, the target spectrum widens to include any animal which has foregut-fermentation, as well as metamorphosing insects and amphibians. Indeed, Lajmanovich et al. [65] show that Bt can target herbivorous tadpoles. This is concerning, given the already stressful environmental conditions that amphibians are encountering. Even those who may be working to diminish the presence of chemical pesticides in the environment by moving to more a “natural” control may be causing unintended damage. For livestock fed on the remnants of Bt-containing crops, the impact on their digestion may cause morbidity and even mortality [59,60,61]. It seems that silaging may break down the Bt toxin [63,64], which is a simple fix for a consequential problem.

There is still a lot of work to be carried out to clarify the impact of latex on digestive systems. In 1989, C.C. Webster and W. J. Baulkwill concluded that “…*the function of latex and rubber in the plant remains unknown*”. [74]. Three decades later, Abarca et al. (2019) stated that *“There is no scientific evidence of the metabolic role of latex in plants*”. [9]. In 1999, Shipley bemoaned “*In addition, virtually all studies comparing anatomy and physiology of browsers and grazers focus on ruminants, and thus fail to consider similar adaptations by other types of herbivores, such as hindgut-fermenters (e.g., rodents, rabbits, horses) and non-ruminant foregut-fermenters (e.g., kangaroos, sloths)*” [13]. But there is progress. Besides plugging wounds, there may be no functional role for latex in the plant itself, except to prevent browsing by herbivores. Additional work to examine the latex-adapted foregut herbivore vs. latex-maladapted hindgut herbivore hypothesis will have an impact on feed and forage provided to livestock and captive animals. Further, providing guidelines for Bt use to minimize off-target impact will benefit foregut-fermenting animals already stressed by environmental or captive conditions.

## 5. Conclusions

In summary, the alkaline anterior chambers of foregut-fermenting herbivores are consistent with being an evolutionary adaptation to the widespread presence of latex in forage. Since latex is liquid in neutral to alkaline pH, but solidifies to rubber in acidic pH, it is latex *itself*, not the additional chemicals it contains, that is the primary herbivory deterrent. Protection from latex coagulation simultaneously makes foregut-fermenting animals more susceptible to the action of alkaline-pH-activated Bt toxin, which is widely used as an insect biocontrol agent.

## Data Availability

The data presented in this study are available in Supplementary Table S1.

## Supplementary Materials

The following supporting information can be downloaded at: https://www.mdpi.com/article/10.3390/life13112195/s1, Table S1: Classes of herbivory-deterrent metabolites produced by plants.
