# Supplementary material for: An Alkaline Foregut Protects Herbivores from Latex in Forage, but Increases Their Susceptibility to Bt Endotoxin
Source: Life (Basel). 2023 Nov 11;13(11):2195. doi: 10.3390/life13112195 (PMC10672702; doi:10.3390/life13112195)
Supplement: Supplementary file 1 [file life-13-02195-s001.zip › life-2696346-supplementary.pdf]

Supplementary Table S1: Classes of herbivory-deterrent metabolites produced by plants

Class of metabolite	Mechanism of action	Comments
Cyanogenic glycosides	R-CH-CN core, derived from amino acids. Molecules break down to release cyanide which is a respiratory poison.	Wide distribution in all vascular plants e.g. cassava. Some insects, such as larvae of the southern armyworm have adapted to cyanide in their diet.
Glucosinolates	>100 variations, derived from amino acids. When released from special compartments, they produce "mustard oils" which produce a pungent taste. Breakdown products in the gut may cause discomfort.	Present in radish, mustard, and cabbage families. Activated when plant cells are broken by the action of the enzyme myrosinase. Cooking inactivates the enzyme. Many pest insects have adapted to feed on plants containing glucosinolates.
Non-protein amino acids	Derived from amino acids. Substitute for biogenic amino acids and get incorporated into proteins which then become non-functional. Their breakdown products may also be toxic.	Diversity of plants, and many legumes contain non-protein amino acids. Examples of non-protein amino acids are canavanine (whose breakdown product is an analog of ornithine) and mimosine which is broken down by gut bacteria to toxic compounds. Resistant insects produce tRNAs that can discriminate against non-protein amino acids.
Alkaloids	Grouped together with true alkaloids, pseudoalkaloids, and protoalkaloids. True alkaloids are derived from amino acids, are basic and have heterocyclic rings with nitrogen. Generally toxic, have psychedelic and bioactive effects, or are unpalatable. May be induced in response to microbial attack (phytoalexins).	Over 15,000 types, produced by 20% of plant species, including all of the poppy family of plants (Papaveraceae). Many alkaloids are bioactive and have been sought after for that activity. Contains a slew of compounds e.g. caffeine (coffee), solanidine (potato), mescaline (cacti), strychnine (*Strychnos* seeds), nicotine (tobacco), cocaine (opium).
Plant phenolics	Secondary metabolites originate from a variety of metabolites including aromatic amino acids, flavonoids, terpenes, tannins, lignin precursors. Deterrence mechanisms are through unpalatability, toxic effects, induction in response to microbial attack (phytoalexins), cause complex formation with and inhibition of enzymes, induction of free radicals, or thickening of cells by deposition.	Phenolics are produced constitutively or induced in response plant damage from a variety of pathogens including fungi, nematodes and insects. In some cases, phenolics, cellulose and latex form indigestible phytobezoars that cause alimentary distress.
Plant terpenes	Antifeedants, toxins, insect developmental interference by sterols, wound-response volatile terpenes that deter insects or recruit natural predators of herbivores. Sesquiterpenoids function as phytoalexins (induced upon microbial damage). Saponins are terpene glycosides and have antifeedant or toxic properties.	Diverse class with more than 40,000 known members built with an isoprenoid (C_5H_8) monomer. Latex contains a variety of terpenoids, in addition to the rubber polymer, some of which can be toxic.
